# Applying Molecular Phenotyping Tools to Explore Sugarcane Carbon Potential

**DOI:** 10.3389/fpls.2021.637166

**Published:** 2021-02-19

**Authors:** Maria Juliana Calderan-Rodrigues, Luíza Lane de Barros Dantas, Adriana Cheavegatti Gianotto, Camila Caldana

**Affiliations:** ^1^Max Planck Institute of Molecular Plant Physiology, Potsdam, Germany; ^2^Centro de Tecnologia Canavieira, Piracicaba, Brazil

**Keywords:** sugarcane, carbon, sucrose, biomass, molecular phenotyping

## Abstract

Sugarcane (*Saccharum* spp.), a C_4_ grass, has a peculiar feature: it accumulates, gradient-wise, large amounts of carbon (C) as sucrose in its culms through a complex pathway. Apart from being a sustainable crop concerning C efficiency and bioenergetic yield per hectare, sugarcane is used as feedstock for producing ethanol, sugar, high-value compounds, and products (e.g., polymers and succinate), and bioelectricity, earning the title of the world’s leading biomass crop. Commercial cultivars, hybrids bearing high levels of polyploidy, and aneuploidy, are selected from a large number of crosses among suitable parental genotypes followed by the cloning of superior individuals among the progeny. Traditionally, these classical breeding strategies have been favoring the selection of cultivars with high sucrose content and resistance to environmental stresses. A current paradigm change in sugarcane breeding programs aims to alter the balance of C partitioning as a means to provide more plasticity in the sustainable use of this biomass for metabolic engineering and green chemistry. The recently available sugarcane genetic assemblies powered by data science provide exciting perspectives to increase biomass, as the current sugarcane yield is roughly 20% of its predicted potential. Nowadays, several molecular phenotyping tools can be applied to meet the predicted sugarcane C potential, mainly targeting two competing pathways: sucrose production/storage and biomass accumulation. Here we discuss how molecular phenotyping can be a powerful tool to assist breeding programs and which strategies could be adopted depending on the desired final products. We also tackle the advances in genetic markers and mapping as well as how functional genomics and genetic transformation might be able to improve yield and saccharification rates. Finally, we review how “omics” advances are promising to speed up plant breeding and reach the unexplored potential of sugarcane in terms of sucrose and biomass production.

## Introduction

Sugarcane (*Saccharum* spp., Poaceae) is a perennial C_4_ grass with an exceptional ability to convert light and nutrients into chemical harvestable energy such as sucrose. It is particularly found in tropical and subtropical climates, in which warm temperatures and water availability underpin maximum growth rates (Moore and Botha, 2014; Hoang et al., 2015). Apart from being the main crop considering harvested tonnage, sugarcane high use efficiency of agricultural supply (e.g., fertilizers and water) and the local processing of its feedstock by the mills render a favorable ratio of total energy output per input required for its production (Lam et al., 2009; Moore and Botha, 2014). The outstanding capacity of biomass accumulation mainly relies on the mature culm, in which up to 64% of dry weight is allocated in the form of sucrose and fiber (cellulose, hemicellulose, and lignin), whereas the rest of dry matter (∼36%) comes from the leaves (Casu et al., 2004; Singh et al., 2010).

**FIGURE 1:**
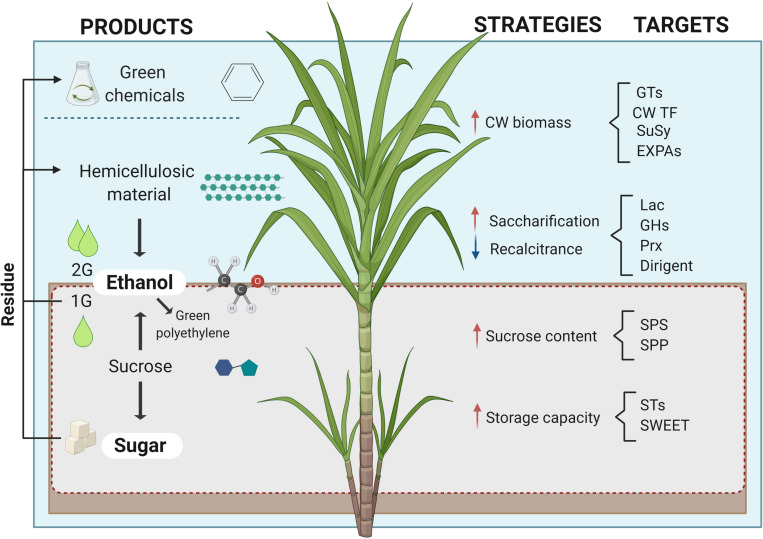
Sugarcane in a biorefinery context. Products, strategies, and molecules to be targeted in molecular phenotyping to achieve sugarcane C potential. Red and blue arrows indicate the desired increase and decrease of the mentioned strategies, respectively. Image created with BioRender.com^2^.

The sugarcane industry is focused on processing the culm for extracting sucrose and its byproducts, molasses and bagasse, used for producing sugar, bioethanol, and bioelectricity (see Box 1). More recently, alternative conversion processes of sugarcane biomass have been developed for manufacturing high-value compounds such as bioplastics, organic acids, molecular hydrogen, and advanced lignocellulosic compounds (Klein et al., 2019). This biorefinery approach would pave the way for the complete use of sugarcane carbon (C) for further improving its sustainable production potential (Figure 1). The emerging paradigm change in sugarcane breeding programs aims to alter the balance of C partitioning as a means to increase the plasticity of the sustainable use of this biomass for green chemistry and/or metabolic engineering. In contrast to sugarcane, the so-called energy cane maximizes growth with greater productive potential by redirecting the metabolism to accumulate more fiber instead of sucrose. The versatile use of its biomass, the existing agronomic and processing infrastructure comprising large-scale operating mills place sugarcane as the leading crop for biomass production and efficient C usage.

**Box 1**

Ethanol 2G is changing the paradigm for biofuel production.

Besides the process of first-generation (1G) ethanol production using sucrose accumulated in sugarcane culms, the bagasse (or the whole plant) can provide extra yield through the saccharification of sugars from the cell wall (CW) polysaccharides, named 2G. CW is the raw material for several green chemistry-derived products and the carbon matrix used for 2G production. Such biofuel manufacturing relies on (1) circumvention of the CW recalcitrant by pretreatment of lignocellulosic material, (2) hydrolysis of the CW polysaccharides such as cellulose, hemicellulose, and pectin, and (3) fermentation of the released sugars that will be distilled to ethanol (Canilha et al., 2012). One of the alternatives to overcome 2G recalcitrance is through the comprehension of CW molecular components and how they are structured. New sugarcane cultivars called “energy cane,” with more proportion of genetic background from the ancestral species (*S. spontaneum*) and increased CW/fiber content, have been developed for this purpose. Since 2G can be produced from the sugar industry residues, this could be a sustainable approach, as well as addressing food security to increase the industrial yield for the same cultivated area. More than a decade of heavy investment in overcoming the technical difficulties of 2G production finally paid off, turning this biofuel into a commercially competitive commodity. Compared to 1G, the addition of the 2G process incremented 50% the production and reduced 35% of C footprint^1^.

### Potential Targets for Improving C Use in Sugarcane

**FIGURE 2:**
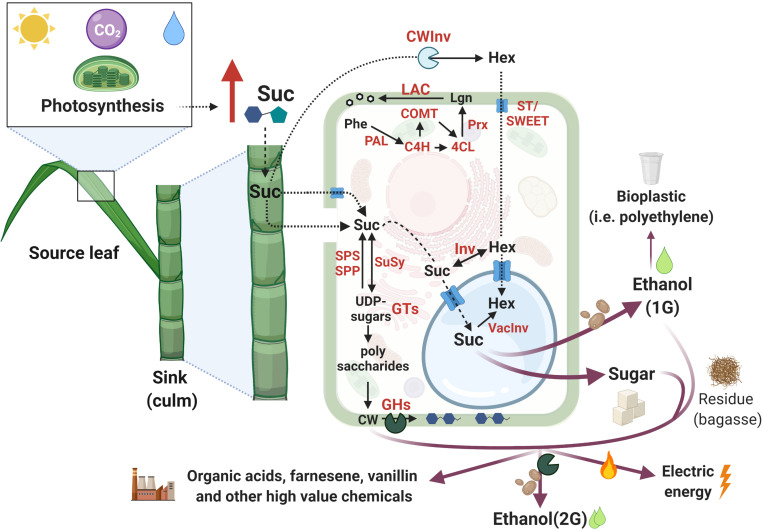
Schematic representation of sugarcane C partition pathways from photosynthesis to the added-value products. Red arrows represent increased content and red letters indicate proteins involved in the mentioned reactions. Pathways were simplified to allow visualization of the main findings in sugarcane. Additional intermediates may not be present. Images are not in scale and were created with BioRender.com^2^. Inspired by Wang et al. (2013).

Photosynthesis represents the only source of C for synthesizing the elementary building blocks of cell structure, and, therefore, the pattern of C assimilation, storage, and utilization are key determinants for biomass accumulation (Smith and Stitt, 2007; Siqueira et al., 2018; Wingler, 2018). During photosynthesis, C assimilation occurs in the source leaves, from where photoassimilates are transported by the phloem and imported into non-photosynthetic C-consuming sink tissues (e.g., roots, culms) providing energy to sustain metabolism (consumption) or support growth (storage) (McCormick et al., 2009; Wang et al., 2013). Sucrose phosphate synthase (SPS) and sucrose phosphate phosphatase (SPP) are two enzymes that take part in sucrose synthesis. SPS converts fructose-6-P and UDP-glucose to sucrose-P, which is then turned into sucrose by SPP. Despite its name, sucrose synthase (SuSy) performs a reversible reaction that *in vivo* preferentially cleaves sucrose to generate UDP-glucose and fructose. The transport of sucrose to sink organs occurs through the phloem and its entrance in the sink cells can be mediated by membrane sugar transporters (ST) (reviewed by Wang et al., 2013; Figure 2). Following storage, sugars can be translocated to the vacuole or fixed into polymers that can be remobilized for energy (e.g., starch) or structural biomass (such as lignocellulose) (Wang et al., 2013).

The great potential of sugarcane biomass accumulation mainly relies on the photosynthetic C partitioning into sugar and fiber in the culms (Whittaker and Botha, 1997; Figure 2). Higher incorporation of C into fiber leads to a reduced C portion available for sucrose storage and vice-versa (Wang et al., 2013). UDP-glucose is the source for different UDP-sugars that are precursors for cell wall (CW) polysaccharides (reviewed by Verbančič et al., 2018). During internode elongation and expansion, almost 50% of C is branched off to cellulose, hemicellulose, and lignin synthesis for CW assembly and, consequently, fiber deposition. However, internode maturation favors sucrose storage in the vacuole (up to 66% of C), while only 8% of the C is diverted into fiber (Bindon and Botha, 2002). In contrast to fiber deposition, sucrose metabolism is very dynamic in sink organs, enabling fast shifts between its supply and demand, increasing sink strength, and minimizing the sugar-mediated repression of photosynthesis (McCormick et al., 2009; Wang et al., 2013).

The high capacity of storing an osmotically active solute in the parenchyma tissue of the culm makes sugarcane a unique source-sink system, particularly compared to most species that store insoluble polysaccharides like starch in terminal sinks (McCormick et al., 2009; Wang et al., 2013). Although enhancing culm sucrose content is one of the primary goals for increasing sugarcane yield, the main constraints in the storage of this disaccharide relies on the source to sink relations and how C is partitioned. Certain physiological processes can restrict sucrose storage such as C allocation into other pools than culm storage, phloem loading in the leaf and unloading in the culm, besides the own culm metabolism including sucrose turnover and transport into the vacuole (Lalonde et al., 2003; Walsh et al., 2005; McCormick et al., 2009). Like other C_4_ species, sugarcane has developed complex biochemical and morphological specializations that bring about superior radiation-, nitrogen (N)- and water-use efficiencies relative to other C_3_ species (Atkinson et al., 2016; Furbank, 2016; Sage et al., 2018; Leakey et al., 2019). Nonetheless, sugarcane’s high productive potential has been questioned as the photosynthetic capacity of most cultivars is reported to reach similar performances to C_3_ species in mature but not in young plants (Sage et al., 2014). The reduced photosynthetic capacity is mainly caused by N depletion in leaves and high accumulation of sucrose in culms. As a consequence, the photosynthetic activity progressively declines along with culm maturation in commercial cultivars, possibly indicating sink regulation of source capacity (McCormick et al., 2009; Wang et al., 2013). This feedback repression can be relieved depending on the sink size and activity. Therefore, strategies to unravel the key players regulating the feedback between source and sink tissues in sugarcane have great potential to enhance sucrose accumulation.

Sucrose metabolism in the culm encompasses a highly plastic network that allows rapid re-synthesis, partitioning to other C pools (like the CW), and turnover between cell compartment and organs depending on physiological and developmental conditions. In contrast, the metabolic pathways involved in fiber deposition are almost irreversible. The synthesis of cellulose, hemicellulose, and lignin is mediated by different metabolic pathways that physically interact to form the highly complex and compact structure of lignocellulose. This polymer network acts as a physical protective barrier from invaders and abiotic stresses, and thus limits the release of its fermentable sugars at the cell surface, whose collapse will ultimately lead to cell death. Particularly during internode maturation, cell elongation and sucrose accumulation discontinue, while CW thickening and lignification takes place (Botha and Black, 2000). Although the non-linear structure of lignin is the major responsible for biomass recalcitrance, this phenolic polymer chemical diversity provides a rich source of high-value products such as vanillin and aromatic compounds, phenols, macromolecules such as C fiber, and hydrocarbons. Understanding the mechanism underlying the tight regulation between sugar and fiber partition in the culm is one of the crucial aspects to unravel the main players in biomass accumulation in this crop.

Lipids, such as triacylglycerols (TAGs), are an alternative form of C storage. However, these lipids are a competing sink for fixed C after photosynthesis, preventing C from being stored as carbohydrates. These fatty acids accumulate in the cytosol as oil bodies that can be metabolized or recycled to form cell structures. TAGs are commercially interesting as a source for biodiesel, presenting the advantage of releasing twice the energy of carbohydrates (reviewed by Zale et al., 2015). Despite that, TAGs have a long way ahead, as breeding strategies have traditionally focused on sucrose or biomass. Moreover, initiatives to increase TAGs accumulation in sugarcane did not surpass oilseed crop yields and were shown to negatively impact on Brix and biomass contents (Parajuli et al., 2020). Despite considerable efforts in conventional sugarcane breeding, sugar yield seems to have reached a plateau due to its narrow germplasm genetic basis and the tradeoff with other traits such as abiotic stress tolerance or biotic stress resistance (McCormick et al., 2009). The complex polyploid nature of sugarcane also hampers the use of molecular breeding tools commonly used for most of the crops. More recently, the advance of molecular phenotyping tools has emerged as an alternative to unravel the complex genetic structure of this crop and gain further insights into physiological and biochemical processes opening perspectives for more sustainable use of C (Figure 1). Although molecular phenotyping has been broadly used for understanding sugarcane responses to environmental cues, biotic or abiotic, in this review we mainly focused on the advances regarding metabolic routes for a positive C balance.

### Sugarcane Requires the Development of New Molecular Breeding Approaches for Targeting Polyploids

Current sugarcane breeding programs are mainly focused on developing cultivars with improved yield (biomass production and/or sugar content) and biotic/abiotic stress tolerance. More recently, the existing genetic diversity has been used for selecting energy cane varieties classified as type I and II. Type I has enhanced both sucrose and fiber content, whereas type II varieties are oriented for increasing fiber content only. The modern sugarcane cultivars are hybrids derived from interspecific crosses, mainly between *S. officinarum* (x = 10; 2n = 8x = 80) and *S. spontaneum* (x = 8; 2n = 5x = 40–16x = 128), but with contributions from other *Saccharum* spp. (*S. barberi*, *S. robustum*, *S. sinense*) (Matsuoka et al., 1999; Cheavegatti-Gianotto et al., 2011). Nowadays, the sugarcane breeding programs still rely on these pioneer hybridizations performed almost a century and a half ago (Matsuoka et al., 2005; Berding et al., 2007; Yadav et al., 2020). Despite contemporary efforts to broaden the genetic basis, limited results were achieved mainly due to the lack of genetic tools needed for tracing the incorporation of advantageous genes into the genetic pool of the noble cultivars (Berding et al., 2007; Moore and Botha, 2014). As a consequence, and despite its irrefutable importance in providing cultivars with high sucrose content and resistance to environmental stresses, breeding seems to have reached a yield plateau in the main sugarcane producing countries (Australia, Brazil, China, India, Thailand, and United States) in the last decade. The narrow genetic base of modern cultivars, the highly complex genetic architectures of quantitative traits, and the very long breeding cycle lengths have been mentioned as the principal factors limiting the release of new cultivars (Yadav et al., 2020).

A typical sugarcane breeding program consists of three major steps: germplasm collection/evaluation, hybridization, and selection (Berding et al., 2007). As sugarcane flowering is photoperiod-induced, all crosses are performed within a narrow window, from late April to July at breeding stations located around a 10° latitude in the South Hemisphere. Crossings are planned based on parental genotypes with the desired characteristics and flowering synchronization. It is well known that several important traits such as, stalk diameter, height and tillering, present inbreed depression while it seems not to be the case for traits related to fiber and sugar accumulation (Verma, 1990; Matsuoka et al., 2005; Anbanandan and Eswaran, 2018; de Paula et al., 2020). Therefore, to promote heterosis breeders prefer selecting non-closely related genotypes when performing such crosses. Besides, to increase the chances of success, tens to hundreds of thousands of seedlings must be generated at each crossing season. Despite the recognized presence of heterosis in sugarcane, breeders have been exploiting the additive genetic portion of the phenotypic variance (narrow-sense heritability) in traditional sugarcane breeding programs (Matsuoka et al., 2005). Once genetic variability is achieved from the initial crosses, the next steps rely on successive and increasingly stringent selections. The whole process lasts around one decade and the rate of success is extremely low. Estimates point out that one commercial variety is obtained after assessing an average of 250,000 seedlings at the germplasm evaluation stage (Cheavegatti-Gianotto et al., 2011). Just recently, de Paula et al. (2020) proposed the development of heterotic groups and the adoption of reciprocal recurrent selection in sugarcane breeding programs to exploit non- and additive effects bringing new possibilities to sugarcane breeding programs.

In many crops, molecular breeding has fostered the selection of desired traits in a cost- and time-effective manner. The complex sugarcane genetic composition of modern hybrids led to polygenic control of important agronomic traits, hindering the development of genetic maps required for quantitative trait locus (QTL) identification and selection of linked markers for assisted selection in breeding programs. Furthermore, the high heterozygosity of hybrids and inbreeding depression restrict the development of mapping populations derived from inbred lines. Yet, the presence of hom(oe)ologs and close paralogs for most of the sugarcane loci, derived from the highly polyploid and uneven genetic background from sugarcane hybrids, deepens the challenge for a successful genotyping.

In contrast to plant species with simpler genomes, segregation occurs at the first generation of a progeny derived from biparental crosses in sugarcane (Pastina et al., 2010). Consequently, the first sugarcane genetic maps of individual parental genotypes could only be assembled after the development of single-dose markers (SDM) (Wu et al., 1992; Al-Janabi et al., 1993; da Silva et al., 1993; D’Hont et al., 1994; Daugrois et al., 1996; Mudge et al., 1996; Guimarães et al., 1999). In this approach, a copy (dose) of a given maker is present in either one or both parents segregating into 1:1 or 3:1 (presence: absence) ratio, respectively, in the mapping population (Pastina et al., 2010). The integration of individual parental maps became possible only after expanding the SMD approach by using simultaneous maximum-likelihood estimation of linkage and linkage phases (Garcia et al., 2006). As single markers do not represent all genetic information of a locus, maps generated with this method only allow partial genomic coverage, around 33–50% (Pastina et al., 2010). Despite this limitation, numerous QTL mapping studies for agronomical traits as yield, sugar production, and pest/disease resistance have been developed for sugarcane (Sills et al., 1995; Daugrois et al., 1996; Hoarau et al., 2002; Ming et al., 2002; Jordan et al., 2004; da Silva and Bressiani, 2005; McIntyre et al., 2005, 2006; Reffay et al., 2005; Aitken et al., 2006, 2008; Aljanabi et al., 2006; Wei et al., 2006; Piperidis et al., 2008; Raboin et al., 2008; Pinto et al., 2010). More recently, models with higher complexity have been applied to QTL studies in sugarcane, accounting for genetic × environment interaction (Pastina et al., 2012), codominant markers (Costa et al., 2016), and genotyping-by-sequencing (GBS) data (Balsalobre et al., 2017).

Despite the advances, mapping studies usually detect QTLs with individually small effects on agronomic traits, reducing their practical application for MAS in sugarcane breeding programs. Several QTLs typically control quantitative traits, each of them with small effects. This is particularly true for complex traits such as biomass production and composition. The small effects on agronomic traits presented by QTLs diminish the possibility of identifying MAS for improving selection performance in sugarcane breeding programs. The exceptions are traits related to disease resistance, as the case for *Bru1* gene, a major dominant resistance locus for the brown rust disease (Daugrois et al., 1996). More recently, other QTLs related to resistance against the orange rust disease have enabled the development of a molecular marker in a F1 segregating population derived from a cross between two hybrid sugarcane clones, CP95-1039 and CP88-1762 (Yang et al., 2018).

Genome-wide association studies (GWAS) are observational studies of existing populations aiming to identify marker-trait associations (MTAs) in genetically diverse populations. One of the requirements for its successful application is the presence of genome-wide linkage disequilibrium (LD), an ideal scenario for modern breeding programs as it relies on relatively recent interspecific crosses and few opportunities for genomic recombination. The estimate of LD in sugarcane is challenging due to difficulties in obtaining a saturated genetic map. Initial work applying amplified fragment length polymorphism (AFLP) markers estimated that hybrids present LD among closely linked markers and that such LD decreased within 0–30 cM window (Raboin et al., 2008). Barreto et al. (2019) demonstrated that LD was strongest among 5 cM, still significant in the first 15 cM, with a clear decay over higher distances. A deep sequencing (∼98 ×) of coding regions of 307 sugarcane germplasm accessions revealed the presence of LD, ranging from 3163.5 to 4451.0 Kbp in sugarcane hybrids (Yang et al., 2018). These latter authors pointed out that the estimate of LD in sugarcane is larger than most of the species analyzed to date, such as *Arabidopsis thaliana* (∼10 Kbp), sorghum (∼150 Kbp), and soybean (∼ 420 Kbp) (Kim et al., 2007; Morris et al., 2013; Zhou et al., 2015). Yang et al. (2019) also pointed out that such high LD extent would not require high marker density to perform GWAS but would difficult gene fine mapping or even map-based cloning studies as it would be difficult to break linkage drag.

Although the construction of genetic maps is not required, GWAS can still be affected by the high heterozygosity of segregating loci underrepresented by the currently available SDM. Barreto et al. (2019) applied GWAS to the Brazilian panel of sugarcane genotypes and successfully found 23 MTAs for soluble solid content, sugarcane yield traits, stalk number, height, and weight with a low phenotypic variation ranging from 1 to 7%. Similar results were obtained for other GWAS of yield or disease resistance traits in sugarcane (Debibakas et al., 2014; Gouy et al., 2015; Racedo et al., 2016; Yang et al., 2020). The high ploidy levels and the quantitative inheritance of the evaluated traits were the main factors explaining the low variability.

Genomic selection (GS) of individuals by their predicted breeding values estimated from genome-wide DNA marker profiles is a strategy to overcome the small effects of individual QTLs and MTAs. The development of GS models uses a training population, in which the individuals are genotyped and phenotyped. Once the GS model is developed, individuals from the prediction population are selected based on their genomic estimated breeding values and genome-wide marker data (Yadav et al., 2020). The feasibility of GS in sugarcane was explored by testing two populations of 167 individuals, each genotyped with 1,488 diversity arrays technology (DArT) markers and 10 traits, including sugar and bagasse contents, digestibility and composition of the bagasse, plant morphology, and disease resistance (Gouy et al., 2013). By evaluating 4 statistical predictive models, namely Bayesian LASSO, ridge regression, reproducing kernel Hilbert space, and partial least square regression, the authors have found median correlations ranging from 0.13 (smut resistance) to 0.55 (Brix) in cross-validation between panels. Similar prediction accuracies (0.25–0.45) were found recently for predicting traits related to C partitioning and allocation such as yield and sugar content (Deomano et al., 2020). Altogether, GS seems a promising approach to be implemented in sugarcane breeding programs to foster the selection of new varieties.

Unlike diploid species, allele dosage can represent a large source of phenotypic variation in sugarcane, which cannot be captured by single-dose markers and the current statistical models. Recent advances have developed mapping models for loci with high allelic dosages (Garcia et al., 2013; Gerard et al., 2018; Pereira et al., 2018; Mollinari and Garcia, 2019). The explicit consideration of allele dosage into QTL, GWAS, and genomic selection models provides a more realistic representation of the genotypic architecture of the sugarcane genome, and consequently enhances the probability of successfully incorporating molecular markers into breeding programs.

### Notable Strategies for the Partial Sequencing of Sugarcane Complex Genome Enabled the Exploration of C Partition Targets

Attempts in understanding the complex genetic structure in sugarcane have achieved considerable advances with new technologies of DNA and RNA sequencing. Sugarcane genetic and phenotypic complexity can be illustrated solely by taking the example of the pioneer crosses between *S. officinarum* and *S. spontaneum*. These species have distinct traits of great potential: sweetness and robustness that aligned gave rise to the increased yield of the current commercial hybrids. *In situ* hybridization (GISH) studies have demonstrated that *S. officinarum* contributed with 80–85% of chromosomes, while *S. spontaneum* contributed with 10–15% of chromosomes to the current commercial lines. Additionally, around 5% of the chromosomes of commercial hybrids are recombinants of *S. saccharum* and *S. spontaneum* chromosomes (Jannoo et al., 1999; D’Hont, 2005). As there are two sets of chromosomes of different species coexisting in the cell nuclei of commercial hybrids, recombination in the sense of crossing over preferably occurs among the chromosomes of the same species (within each set), as they are more similar and therefore more likely to be paired with each other during meiosis. Taken together, all those components created a hybrid and both poly- and aneupolyploid large (∼10 Gb) genome (Le Cunff et al., 2008; Thirugnanasambandam et al., 2018) that challenges analysis, as genes at the same loci might be functionally divergent and demand the allele investigation as if they are different genes (Bolger et al., 2017). Not surprisingly, the cultivars’ genomes are yet to be fully covered by sequencing, which demonstrates the disparity between economic relevance and genomic advances, as this potent and complex genetic combination enabled sugarcane to be the source of 80% sugar (International Sugar Organization (ISO), 2020) and around 25–30% ethanol fuel (Renewable Fuels Association (RFA), 2020; Organisation for Economic Co-operation and Development (OECD), 2020) production worldwide. Even though sucrose production per hectare is still one of the most desirable traits in sugarcane breeding, there are only a limited number of identified genes involved in this sugar metabolism.

Despite the significant challenges imposed by the complex structure of the sugarcane genome, many efforts for sequencing and annotating genes accomplished essential advances. The initial step came from the expression sequence tags (ESTs) collection (SUCEST) that provided around 238,000 high-quality sequences from multiple sugarcane organs and tissues (Vettore et al., 2003), followed by several partial sequencing studies (Tan et al., 2013; de Setta et al., 2014; Grativol et al., 2014; Okura et al., 2016; Miller et al., 2017). Gene-enrichment approach using methyl filtration sequencing allowed the coverage of 97.2% of the sugarcane ESTs and 98.4% of sorghum coding sequences (CDS), providing valuable information on single nucleotide polymorphisms (SNPs) from genes associated with sucrose and starch metabolism, identifying the contribution of the two sugarcane ancestor species (Grativol et al., 2014). Furthermore, this analysis confirmed the contribution of *S. officinarum* through non-synonyms modifications and unique SNPs in genes related to sucrose metabolism that could be used for early molecular phenotyping.

Advances in genomic sequencing initiatives, mainly by bacterial artificial chromosome (BACs), were applied to R570 cultivar^3^ to meet upcoming breeding demands (D’Hont et al., 1996; Dufour et al., 1997; Rossi et al., 2003; Vilela et al., 2017). The high adaptability of this cultivar makes it a good hybrid line model for sugarcane. A first initiative enabled to cast a light on the haplotypes and dosage of selected genes related to growth, such as leafy (LFY), phytochrome C (PHYC), and target of rapamycin (TOR) kinase, highlighting both conservation of gene content and synteny with sorghum and rice, besides the pervasive presence of transposable elements (Vilela et al., 2017). The R570 monoploid genome was then extensively sequenced and 25,316 protein-coding gene models were annotated using the principle of gene synteny and the sorghum genome as reference (Garsmeur et al., 2018). This monoploid reference genome offers an excellent opportunity not only to explore hom(oe)ologous allelic variation but also to provide important insights on gene characterization, parental allele prevalence, and potential gene or loci regions associated with C partitioning, biomass, and yield-related traits. This is an interesting and cost-reducing approach for sequencing a vast and complex genome like those of sugarcane hybrids.

Synthetic long-read sequencing technology has opened the perspectives for the whole genome assembly of the commercial cultivar SP80-3280 (Riaño-Pachón and Mattiello, 2017; Souza et al., 2019a), allowing the annotation of 373,869 new genes and their potential regulatory regions. As a proof of concept, the authors investigated two important gene families, SuSy and phenylalanine ammonia-lyase (PAL), related to C partitioning and biomass (Souza et al., 2019a). In contrast to the 5 SuSy genes in sorghum, 43 SuSy CDS clustered in 3 phylogenetic groups were identified in the SP80-3280 assembly, suggesting that the broad diversity of this family might reflect several functional roles based on tissue-specificity, developmental stage, and environmental conditions. Such diversity was confirmed by combining publicly available gene expression studies and *in silico* evaluation of transcription factor binding sites in the regulatory sequences of this family. This combined approach unraveled regulatory patterns associated with circadian regulation or flowering time (Souza et al., 2019a). Similar findings were obtained for PAL, which codes one key enzyme controlling lignin synthesis through the phenylpropanoid metabolism. Whole-genome sequencing approaches allowed to describe the intricate genome of a commercial sugarcane cultivar, finding genes, or genetic regions of metabolic routes important for high-value products to generate new molecular markers, GWAS, or direct targets for breeding programs.

Commercial sugarcane lines have the theoretical potential of accumulating 25% more sucrose (Grof and Campbell, 2001). This potential is suggested to be limited mainly by storage and C loss by respiration. So, the study of sucrose transporters (STs) is a promising alternative toward the achievement of sucrose storage potential. The haploid genome of *S. spontaneum* AP85-441 identified 123 STs clustered in 9 subfamilies and 361 disease resistance genes distributed in 4 rearranged chromosomes. A gene family expansion for tonoplast sugar transporters (TSTs) and tandem duplication-caused expansion of polyol transporter (PLT) and STs families are proposed, placing TSTs as promising candidates to achieve higher vacuolar sucrose accumulation in sugarcane culms (Zhang et al., 2018).

Phylogenetic analysis based on 64 accessions of this species shows three different origins: (1) China, the Philippines, Indonesia, and Papua New Guinea; (2), and (3) India, Pakistan, and Iran (Wang et al., 2008). After several generational rounds of balancing selection and accumulated polyploidization, the rearranged areas might be closely related to features such stress resistance and/or habitat adaptation and, ultimately, to the *S. spontaneum* fitness. Accordingly, the *S. spontaneum* genome shows that 80% of the site-encoding genes have a potential role in biotic stress and, therefore, represent a source of new or updated molecular markers as well as GWAS targets (Zhang et al., 2018). The increasing awareness of predicting the impacts of climate change scenarios on plant yield made genes associated with stress resistance even more relevant (Boyles et al., 2019).

Genome-wise, it is possible to link C partition into valuable compounds and biomass to several genomic regions and specific genes, like those associated with sucrose accumulation (Suman et al., 2012), specifically SuSy (Coleman et al., 2009), STs (Zhang et al., 2018), lignin-related (Kasirajan et al., 2018), and stress tolerance genes (Wang et al., 2008; Zhang et al., 2018). The available genomic data from ancestors and modern cultivars will better support multi-comparative genome studies targeting specific allelic and regulating regions (Bolger et al., 2019).

### Transcriptional Specific Patterns of Sugarcane Genotypes and Organs Enrich Molecular Phenotyping Strategies

Transcriptomes are a valuable resource to identify the expression behavior of a particular gene in different genotypic backgrounds, especially in a complex polyploid and hybrid system as sugarcane (Correr et al., 2020). Also, transcriptomics can be useful for phenotyping genotypes in different developmental stages and environments (Nishiyama et al., 2014), besides evaluating tissue and organ-specific gene expression patterns. The sequencing of sugarcane cDNA and small RNA libraries from internodes in different elongating stages revealed miRNA target genes involved in N, zeatin, and hormone metabolism. As internode elongation is a relevant process that affects growth and biomass, down-regulation of cytokinin and auxin-related genes in the later elongation stages could be explored for increasing the culm biomass (Qiu et al., 2019).

The analysis of gene expression under a set of environmental and management conditions provides effective information on the local development or selection of regionally adapted cultivars. The climate change scenario also brings high CO_2_ levels to the atmosphere, which can positively impact yield taking into account the CO_2_-concentrating mechanisms evolved in C_4_ species. For example, when the commercial cultivar SP80-3280 was submitted to increased [CO_2_], a larger C uptake was observed and plants showed 30 and 40% increment on photosynthesis and accumulated biomass, respectively. Increased CO_2_ levels up-regulated the expression of genes associated with various metabolic pathways such as photosynthesis, sugar transport, and protein metabolism, whereas a few genes related to stress responses were down-regulated (de Souza et al., 2008). Despite the better performance of sugarcane compared to other grasses, the authors highlighted the importance of further exploring elevated CO_2_ scenarios under field conditions.

The expression of genes related to sugar metabolism is a crucial target for phenotyping, as mentioned for genomic approaches. A recent study on different organs of the ancestors *S. officinarum* and *S. spontaneum* shows that the expression of *SuSy* and *STs* is tissue-specific with an expected overrepresentation in sink organs associated with sucrose accumulation (Coleman et al., 2009; Thirugnanasambandam et al., 2019). Correr et al. (2020) observed the up-regulation of *SPS*, *SPP*, and, curiously, *SuSy* expression in sugarcane cultivars with low biomass. Varieties with low sucrose content showed *SuSy* and *SPS* expression induced by the plant hormone ethylene, which is thought to increase the sink strength, and appears to interplay with abscisic acid and sugars to regulate sucrose production in sugarcane (Chen et al., 2019). A comparative analysis between *S. officinarum* and *S. spontaneum* revealed organ-specific *SPSs* and their differential expression patterns. In leaves, a particular *SPS* is suggested to shift the reaction to sucrose production, while in culms the production of either UDP-sugars (preferably) or sucrose seems to be more complex and regulated by diverse SPS, a pattern confirmed by proteomics (Bedinger and Collins, 2018; Ma et al., 2020). The sugars will eventually be exported transporters (SWEET) gene family is an example of well-characterized STs in sugarcane associated with the transport of hexose and/or sucrose across cell membranes (Chen et al., 2010, 2012; Lin et al., 2014). Twenty-two full-length *SWEET* genes were identified in *S. spontaneum* BAC sequences and their highly differential expression evaluated under different conditions, tissues, cells, and developmental stages in both ancestor species *S. officinarum* and *S. spontaneum*, uncovering essential roles in sugar transport, sink accumulation, and even in starvation (Hu et al., 2018). The down-regulated expression of *SWEET* genes was related to low biomass varieties and showed a genotypic-specific expression (Correr et al., 2020).

The transcriptome analysis of a *S. officinarum* x *S. spontaneum* F2 segregating population highlights the correlation between high biomass yield and increased C assimilation in source tissues (Singh et al., 2018). Indeed, leaf transcriptomes show an overrepresentation of genes associated with chlorophyll biosynthesis, light harvesting pathways, and carbon fixation (Calsa and Figueira, 2007), whereas genes associated with sugar transport and CW synthesis are more represented in sink tissues with sucrose accumulation (Casu et al., 2015). Interestingly, a significant proportion of transcripts expressed in leaves displays a rhythmic behavior under circadian conditions (Hotta et al., 2013), connecting C_4_ photosynthesis with the circadian oscillator. The analysis of the rhythmic organ-specific transcriptome of field-grown sugarcane cultivar SP80-3280 highlights the pervasive connection between the sugarcane oscillator and genes associated with sucrose metabolism (Dantas et al., 2020). The importance of the circadian oscillator gene expression regulation in phenotyping relies on data showing that the synchronization between the oscillator and environment enhances plant fitness (Dodd, 2005).

The discovery of genes associated with the lignin content is also a target for phenotyping (Bottcher et al., 2013; Vicentini et al., 2015; Ferreira et al., 2016; Kasirajan et al., 2018), as this polymer is one of the causes of the recalcitrance of 2G ethanol production. Transcriptomic data from two lignin contrasting sugarcane genotypes, IACSP04-529 (high content) and IACSP04-683 (low content), both developed at the IAC^4^, revealed that genes associated with lignin biosynthetic pathway are expressed in a developmental and tissue-specific manner in the culms. Genes associated with S-branch pathway were up-regulated in inner stem tissues during early stages of the plant development, associated with developmental lignification (Bottcher et al., 2013). This data is supported by another study also featuring contrasting genotypes on lignin content that show differential expression of over 2,000 transcripts, many involved in lignin metabolism, expressed in internodes of commercial hybrids IACSP04-065 (low lignin content) and IACSP04-627 (high lignin content) (see text footnote 2) (Vicentini et al., 2015). The transcriptional comparison between mature and immature sugarcane tissues of two groups containing 10 genotypes each with differential fiber content highlights contrasting expression of genes related to both cellulose and lignin biosynthesis. Ultimately, the expression patterns observed in genes associated with CW development in immature tissue, regardless of the genotypes analyzed, point toward more targets for lignin-oriented phenotyping (Kasirajan et al., 2018). Comparatively, transcriptomic data from ancestral sugarcane species *S. officinarum*, *S. spontaneum*, and *S. robustum* and the commercial hybrid RB867515, developed at the RIDESA in Araras^5^, highlights an overrepresentation of genes for lignin biosynthesis in *S. spontaneum* such as *pal* and *4-coumarate:CoA ligase* (*4cl*). Both RB867515 and *S. officinarum* clustered in all analyzed source and sink organs regarding lignin-associated transcripts (Ferreira et al., 2016), which was expected, given the high impact of *S. officinarum* in the commercial line genome (Jannoo et al., 1999; D’Hont, 2005). The data also showed 18 transcription factors likely related to CW biosynthesis, including members from the NAC (*ScNAC36* and *ScNAC83*) and MYB families (*ScMYB52*) (Ferreira et al., 2016). Plus, a transcriptional analysis of dirigent proteins (DIR), thought to take part in lignin metabolism, has placed them in secondary CW maintenance, which could involve sugar accumulation in sugarcane culms (Nobile et al., 2017).

Transcriptomes are also important for bringing up data on expressed haplotypes. The analysis of haplotypes associates parental-inherited patterns that seem to pass along rounds of crosses through breeding, even though recombination does not seem to be equally distributed in chromosomes (Akhunov, 2003; Bevan et al., 2017), as it might be the case for other polyploid crops (Borrill et al., 2018). Data from the updated cDNA libraries for both parental species *S. officinarum* and *S. spontaneum* and the hybrid cultivar SP80-3280 suggest that the main traits from each ancestor associated with sucrose accumulation and stress tolerance are linked to differential gene and allele expression, expressed haplotypes, and alternative splicing (Nishiyama et al., 2014). Data from the same cultivar showed that alternative splicing correlates with the temperature of the naturally fluctuating field environment when winter and summer plants are compared for the splicing profile of the central oscillator homologs in sugarcane (Dantas et al., 2019). This might suggest that different mechanisms of gene expression regulation are responsive to the environment on diverse levels, considering that both alternative splicing and the circadian oscillator are gene expression regulatory networks and, as such, responsive to the plant surroundings.

Besides the increased expression of photosynthetic-related genes, high biomass genotypes also presented more up-regulated CW-related transcripts (Singh et al., 2018). Accordingly, sugarcane high biomass genotypes showed up-regulation of genes related to xylan metabolism and Golgi (Correr et al., 2020). Xylan is the major hemicellulose in grasses CWs (Vogel, 2008) and the Golgi is where the synthesis of CW polysaccharides occurs, mainly performed by glycosyltransferases (GTs) (Verbančič et al., 2018). Moreover, transcriptomic data shows that sugarcane higher biomass accumulation could be associated with the up-regulation of cellulose metabolism, lignin synthesis, callose pathways, expansion genes, and pectin degradation. The increased dry weight in those plants was positively correlated with glucose levels and the fast conversion of sucrose to UDP-sugars, the main precursors of CW polysaccharides (Wai et al., 2017).

As the expected trade-off that takes place on C partitioning, sugarcane genotypes that accumulate more biomass have a higher content of cellulose, hemicellulose, and lignin, and lesser levels of sucrose (Hoang et al., 2017). Progress on gene networks for selecting sugarcane genotypes that accumulate more sucrose or CW biomass relies on the potential identification of gene clusters and associated regulators, as this trait is rather multi-gene and network-oriented. For example, the co-expression network that bridges the expression of transcripts from the lignin biosynthetic pathway to phenotypes of ancestors *S. officinarum*, *S. spontaneum*, and *S. robustum* and hybrid cultivar RB867515 unveiled two families of transcription factors associated with the regulation of CW metabolism in sugarcane. That resonates, for example, with a high correlation between the lignin transcripts and *S. spontaneum*, in agreement with this species phenotype (Ferreira et al., 2016). Co-expression networks like this are important to identify genes associated with a pathway or its regulation, pointing to promising new markers for C distribution into valuable compounds.

Expression atlas is trending as useful overviews on the expression and/or regulatory networks generated from experiments under specific environmental conditions. Such collection helps to understand which genes are co-expressed across different organs, developmental stages, and genotypes, identifying better candidates for selection (markers) of multigenic traits that might be under diverse regulators. Sugarcane commercial cultivar SP80-3280 is an excellent candidate for developing a gene expression atlas due to the number of transcriptome projects done in a plethora of environments and organs, in both microarray and RNA-Seq platforms (Hotta et al., 2013; Cardoso-Silva et al., 2014; Nishiyama et al., 2014; Vicentini et al., 2015; Dantas et al., 2020). Such an initiative can build a great expression collection for selecting traits associated with C gain improvements for molecules of interest.

### Proteomics as an Alternative for Understanding the Cell Wall Recalcitrance

Proteomics offers some advantages over genomics, as it covers the final product of the gene expression (proteins), enrich the number of differential signatures for a given condition (Zhang et al., 2014), and can further provide additional information about the post-translational modifications (e.g., phosphorylation) that are decisive determinants of protein functional roles. Events such as stability, half-life, modifications, and degradation rates do not allow a strict correlation between DNA and protein levels in polyploid species (Islam et al., 2003). However, they support proteomes to be a better source for marker selection because they may target which allele will be more successful to generate the desired phenotype. Mutual information across data layers can be recovered by proteogenomics, which provides a new class of markers gathering data from genomics and proteomics, some of them successfully established in medicine and in progress for plants (Castellana et al., 2008). However, protein quantification methods are not as straightforward as the ones applied for transcripts, which is a challenge specially for the highly polyploid sugarcane.

Sugarcane lignocellulosic biomass has great potential not only for the production of second-generation biofuels, but also for a vast range of high-value compounds. One of the major bottlenecks preventing the use of this feedstock is the recalcitrance to enzymatic hydrolysis due to the multi-scale structure of the plant CW. In contrast to sucrose that can be promptly fermented and converted into ethanol, CW cellulose and hemicelluloses are required to go through a previous hydrolysis step to be transformed into simple sugars prone to alcoholic fermentation (for details see Box 1). Reduced pectin, lignin, and structural protein contents, besides a differential hemicellulose composition, are amongst the specificities of the type II CWs, like the one from sugarcane, in comparison with mostly dicots type I CW (Carpita, 1996). Proteomics offers a unique chance to unravel this intrinsic nature of CW and characterize plant endogenous enzymes prone to efficiently hydrolyze sugarcane CWs. However, CW proteomics was only possible after the development of dedicated protocols focused on the recovery of proteins from this cellular location otherwise discarded in total protein extraction methods.

By adapting protocols designed for the model species *Arabidopsis thaliana* and using different sugarcane tissues at different developmental stages, 283 CW proteins were identified, mainly represented by proteins related to carbohydrate metabolism (PACs) and oxidoreductases (OR) (Calderan-Rodrigues et al., 2014, 2016, 2019; Fonseca et al., 2018). Amongst PACs, 60 glycosyl hydrolases (GH) (Calderan-Rodrigues et al., 2019) that perform CW polysaccharide breakdown during cell expansion and development were identified. To produce 2G, enzymatic cocktails mostly composed of GHs make the CW sugars more accessible to the fermentation step. CW recalcitrance is suggested to be caused by the porosity of the pectin matrix that enables the access of enzymes with a limited size, the interaction between polymers and their association with hemicellulose that determine GH role, the wall structural architecture, and cellulose crystallinity. Substantial progress has been made in this field by using fungal or engineered enzymes, however, the costs and need to scale up the production still lack advances (reviewed by Tavares et al., 2015).

Monocots present a higher percentage of GHs from family 17 compared with dicots, as type II CWs have hemicelluloses primarily composed of mixed (1,3)(1,4)-β-D-glucans (Minic and Jouanin, 2006), which is the substrate for a great deal of GH17 identified in the CW proteomes (Calderan-Rodrigues et al., 2019). The use of GHs on functional studies and genetic manipulation was suggested (Calderan-Rodrigues et al., 2014, 2016, 2019; Fonseca et al., 2018), especially focusing on plant glucosidases and xylosidases that could increment ethanol production similarly to the use of the maize GHs (Han and Chen, 2011).

Grandis et al. (2019) used sugarcane root aerenchyma development as a model for CW self-disassembled cells to map the repertoire of GHs and other enzymes involved in CW degradation. Time-course analysis of the proteome of samples taken along the development of aerenchyma unraveled that this process begins with pectin disassembly by acetylesterases, endopolygalacturonases, β-galactosidases, and α-arabinofuranosidases. A second step aims at the hydrolysis of β-glucan/callose in parallel to the modifications of hemicellulose and cellulose by α-arabinofuranosidases, xyloglucan endotransglycosylase/hydrolases, and expansins (EXPAs). The degree of saccharification was correspondent with GHs enzymatic activity that provided less or more access to the CW sugars, and recalcitrance increased following aerenchyma development. In another survey, Tavares et al. (2018a) showed that in sugarcane aerenchyma, pectin is hydrolyzed by an endopolygalacturonase (also a GH) at the homogalacturonan in middle lamella in an ethylene-dependent manner. Altogether, these enzymes are promising candidates for functional studies in a timely-controlled expression to produce “self-degradable” sugarcane plants, or to be structurally characterized and mimicked to be part of more efficient enzymatic cocktails especially dedicated to hydrolyze sugarcane CWs, having the advantage of reducing the need for chemical pretreatments and thus costs. Accordingly, the overexpression of an arabinofuranosidase (a GH) augmented cellulose content and saccharification efficiency in rice (Sumiyoshi et al., 2013).

Lignin is a phenolic compound from secondary CWs, fundamental for plant structure and development. Together with some polysaccharides like xylan and hemicelluloses, it is the major cause of the plant CW recalcitrance to 2G production (Cesarino et al., 2013; reviewed by Tavares et al., 2015), as mentioned. The lignified portion of sugarcane CW varies in content and composition according to tissue, organ, and developmental stage (Bottcher et al., 2013). Class III peroxidases (Prx) take part in lignin formation and structure (Vanholme et al., 2010) and can act both on promoting the cross-linking of other CW components or weakening them to favor expansion (Passardi et al., 2004). Thus, the alteration of Prxs may reduce wall recalcitrance. Around 43 sugarcane Prx were identified by mass spectrometry (Calderan-Rodrigues et al., 2019), including some predicted to be involved in lignin polymerization such as the Arabidopsis putative orthologs AtPrx52 (Fernández-Pérez et al., 2015), AtPrx64 (Lee et al., 2013), and AtPrx72 (Herrero et al., 2013). Three sugarcane peroxidases showed specific activity profiles based on the tissue analyzed and are good candidates for functional investigation, especially the ortholog of AtPrx52, highly expressed in young and lignified parts of the culm (Cesarino et al., 2012). Other interesting targets proved to reduce lignification and improve saccharification without harming plant development are laccases (LAC) (Le Bris et al., 2019). These enzymes could be used to adjust the polymerization levels, reduce the content or change lignin structure to optimize lignocellulosic deconstruction and result in more yield from biomass-derived products (Calderan-Rodrigues et al., 2016). A promising candidate is SofLAC, a sugarcane laccase able to oxidize monolignols during lignin synthesis (Cesarino et al., 2013).

Salvato et al. (2019) identified sugarcane nuclear proteins in response to water stress that were CW-enriched, mostly having reduced abundance, which can be reasonable when the plant is thriving to survive. These proteins were related to biosynthesis, organization, and secondary wall deposition, such as cellulose synthases and GTs, and are interesting targets to be approached in breeding programs to increase sugarcane CW sugar content for 2G production, especially for developing energy cane new cultivars. Interestingly, tolerance to water stress was also related to the accumulation of a SuSy and higher abundance of proteins related to growth and carbohydrate metabolism in sugarcane roots (Pacheco et al., 2013).

Physiologically, CWs are the largest carbon sinks, and thus the process of C assimilation into sucrose and CW synthesis is associated (Verbančič et al., 2018). Not surprisingly, both the lignin and CW sugarcane genetic programs have been strongly correlated to sucrose synthesis (Papini-Terzi et al., 2009). Therefore, the manipulation of one biosynthetic pathway can impact both 1G and 2G production. Additional N supplementation can accumulate proteins linked to C metabolism in sugarcane. Increased tricarboxylic acid cycle (TCA) enzymes suggested both higher C flow and Susy abundance, indicating accumulation of sucrose in sugarcane stalks under N addition (Salvato et al., 2017), and are pathways that can be engineered into C profitable plants. Biomass generation requires sufficient supply of N, as a large proportion of this nutrient is required for the operation of the photosynthetic machinery, allowing CO_2_ assimilation by photosynthesis. N is also part of the structure of many metabolites including amino acids-building blocks of proteins-, nucleotides and chlorophyll. Therefore, the balance between the C and N metabolism is one of the key determinants for growth and development in plants (Nunes-Nesi et al., 2010). In general, C_4_ species have an improved N use efficiency (NUE) thanks to a reduced photorespiration. In sugarcane, however, C and N balance might differ from other well-studied model plants due the unusual prevailing sucrose metabolism (Robinson et al., 2014). While N depletion is one of the causes for reducing photosynthetic activity in mature leaves, there is not a consensus about the positive impact of N supplementation in sugarcane yield. N supplementation seems also to decrease sucrose content and Brix in several sugarcane varieties (Muchow et al., 1996; Zeng et al., 2020). Nevertheless, it has been reported that N fertilization in cane and ratoons with reduced tillage system can increase stalk number and sugar content (Fortes et al., 2013). It remains to be explored how N fertilization along sugarcane development as well as the NUE of different genotypes can impact sucrose metabolism and biomass accumulation.

In terms of optimizing 2G yield, sugarcane proteomics unraveled information useful for (*i)* designing new varieties with reduced recalcitrance by lowering lignin content or changing its structure by manipulating Prx and LAC (Cesarino et al., 2012; 2013; Calderan-Rodrigues et al., 2019; Grandis et al., 2019); (*ii)* generating plants that are more accessible to hydrolysis and need less biochemical treatments or improving the enzymatic cocktails by using plant GHs (Tavares et al., 2015; Calderan-Rodrigues et al., 2019; Grandis et al., 2019); (*iii)* developing cultivars with increased CW and fiber content by overexpression of glycosyltransferases and cellulose synthases for energy canes (Salvato et al., 2019). Moreover, EXPAs and extensins, besides proteins with domains of unknown functions, are proteins-to-watch that could be useful for breeding programs dedicated to 2G production.

### Metabolomics From the Elucidation of the Metabolic Network Underlying C Use to the Identification of Biomarkers for Breeding

The network controlling plant growth and fitness is largely dependent on metabolism, which is sustained by biochemical reactions through which metabolites are exchanged with the environment to fulfill several cellular and physiological tasks (Basler et al., 2018). Not surprisingly, several important traits, such as biotic and abiotic stress resistance, postharvest processing, and nutritional value, are largely dependent on the metabolic content (Fernie and Schauer, 2009). More recently, it has been shown that more complex traits such as biomass can also be associated with a combination of metabolites (Meyer et al., 2007). Therefore, the study of metabolites, metabolomics, has emerged as a powerful molecular phenotype tool enabling the assessment of the chemical composition of a cell and directly reflecting its physiological status. Thus, investigating the genetic and biochemical basis underlying plant metabolism and their contribution to metabolic diversity can bring important gains in phenotypic traits that rely on metabolic content or dissecting new players in a metabolic pathway (Fernie and Schauer, 2009).

Similar to other crops, many yield traits in sugarcane are related to metabolic pathways involved in C partitioning of sucrose to fiber or optimization of metabolic routes generating novel added-value compounds. Despite the potential of metabolomics in molecular phenotyping, currently, there are only a few studies in sugarcane. One of the pioneer studies aimed at investigating whether changes in the metabolic content were associated with sucrose accumulation along with internode development (Glassop et al., 2007). The gradient of sucrose accumulation along the internode maturation correlated with other sugars like raffinose, trehalose, maltose as well as tartaric acid. Interestingly, the TCA cycle and amino acids were among the changes detected in the immature internode (M2: meristem to internode 2), suggesting that the metabolism is sustaining cell division and elongation by fostering the biosynthesis of building blocks of the cells as amino acids for protein synthesis.

Culm maturation and source to sink relationships were also investigated by the functional characterization of transgenic sugarcane with altered expression of *GAI*, a component of gibberellin signaling pathway and repressor of DELLAs (Tavares et al., 2018b). Changes in the expression levels of *ScGAI* negatively correlated with internode elongation. Metabolic profiling analysis revealed that the differences in culm development of transgenic lines, overexpressing or silencing *ScGAI*, were mainly attributed to changes in C allocation. Whereas the leaves of the ScGAI silencing lines did not display significant changes in their metabolic pool, the source tissue in *ScGAI* overexpressing lines presented lower photosynthetic rates, sucrose, and starch content. Interestingly, the *ScGAI* silencing lines seem to invest more C allocation into the immature internode in the culm, represented by changes in the levels of phenylpropanoids, sucrose, trehalose, galactinol, and myo-inositol. Taken together, the data suggested a role of *ScGAI* interconnecting culm development and primary metabolism, providing a good example of how metabolomics could help elucidate gene function.

Bud outgrowth is a key parameter in plant yield and in sugarcane this trait plays an even more important role not only due to tillering but also due to its vegetative-propagation nature. Axillary buds are naturally in a dormant status, which seems to be partially determined by the culm metabolism (Boussiengui-Boussiengui et al., 2016). To identify the key players and metabolic signatures in this process, a targeted primary metabolism profiling analysis was performed in culms and buds of 16 sugarcane relevant genotypes for breeding in Brazil. The analysis explained that depending on the C demand, the culm can become a source providing energy and C skeleton (e.g., amino acids) for the biosynthesis of building blocks like proteins, enabling the commitment to a developmental transition in bud outgrowth activation. Furthermore, the authors reported that the levels of certain metabolites correlated with sprouting rate. Altogether, data suggested that apart from the identification of metabolic pathways associated with certain agronomic traits (e.g., sprouting), metabolites could be indicators of the genetic potential (Ferreira et al., 2008). Such findings open perspectives for the use of metabolites to predict certain agronomic traits, particularly suitable for complex traits such as biomass accumulation (Keurentjes et al., 2006; Lisec et al., 2007, 2009, 2011; Meyer et al., 2007; Kliebenstein, 2009; Meyer et al., 2010) as it has been already shown in *Arabidopsis* and maize. If this strategy turns out to be well succeed in sugarcane, metabolites could be used to predict plant performance, resulting in effective shortening time and costs for developing new sugarcane varieties.

As it was pointed out, the complex and diverse structure of lignin is one of the main contributors to biomass recalcitrance. The elucidation of the metabolic routes involved in lignin deposition is crucial for determining the main constraints in recalcitrance. Mass spectrometry-based metabolomics has also been used to provide further insights into phenylpropanoid pathway and monolignol biosynthesis using two contrasting genotypes for lignin deposition, enabling the identification of 35 compounds (Bottcher et al., 2013). The analysis revealed that young internodes tended to accumulate more monolignol intermediates, hydroxycinnamates, and hydroxycinnamoyl esters, whereas mature internodes are more prone to accumulate lignin oligomers. Not surprisingly, the sugarcane genotype with higher lignin content also accumulated more lignin oligomers. By using this dataset associated with the EST database, the authors could map key players in lignin biosynthesis that could be used as a reference for further studies in sugarcane biomass recalcitrance.

Synthetic biology has also emerged as a key strategy to enlarge the biochemical context within an organism by modifying its endogenous metabolic network for the biotechnological production of certain value-added compounds (Erb et al., 2017). Sugarcane has also been considered a promising model for the use of plants as a bio-factory for transforming its biomass into a range of energy and high-value chemical products. Apart from its huge capacity of biomass accumulation, the existing infrastructure for sugarcane cultivation, harvesting, transportation, and processing offers an advantage over other bioenergy crops. Recently, the production of the thermoplastic biodegradable polymer polyhydroxybutyrate (PHB), usually obtained from microbial fermentation, has been achieved in biomass crops, including sugarcane (Poirier et al., 1992; Purnell et al., 2007; Petrasovits et al., 2012; McQualter et al., 2014). PHB biosynthetic pathway relies on three enzymes, which use acetyl-CoA as a substrate. Although the chloroplast-targeted PHB pathway from *Cupriavidus necator* resulted in phenotypically normal sugarcane plants, the accumulation of PHB triggered C starvation-symptoms, including a decrease in biomass accumulation, probably due to a diversion of C and energy to support PHB synthesis (Petrasovits et al., 2012; McQualter et al., 2014). To identify the major metabolic constraints in using sugarcane to produce PHB, McQualter et al. (2016) performed a systems biology approach coupled with metabolic modeling. By assessing the dynamic of the metabolites and certain physiological parameters, the authors found that the transgenic PBH-producing sugarcane displayed only mild C-deficiency caused by a decrease in C assimilation as well as sucrose and starch in the leaves. The metabolic model predictions supported by the experimental data indicated that a disruption in ATP supply in the bundle sheath cells is caused by the presence of PHB granules, which interfere with the photosynthetic machinery (McQualter et al., 2016). By using a system approach associated with modeling, the authors could suggest some tradeoffs in the production of PHB, such as the exclusion of PHB of bundle sheath cells, or production in other organelles, which could be used as strategies to improve this high-value compound in sugarcane, optimizing the biomass use (McQualter et al., 2016).

### Biotechnology as an Emerging Approach for Improving Sugarcane Variety Performance in a Gene-Specific Manner

One of the ultimate goals of molecular phenotyping is to discover promising candidate genes that could be targeted to improve certain traits into commercial varieties using recombinant DNA technology. A major advantage is the integration of genes or even traits that do not belong to the natural pool of the *Saccharum* germplasm into sugarcane breeding programs. Apart from improving the performance of traits like sucrose accumulation, this technology is particularly relevant for the introgression of missing traits in highly productive cultivars, such as insect resistance, herbicide tolerance, or new metabolic pathways for the synthesis of high-value products.

In addition to the genome complexity, its semi-perennial and vegetative propagation nature, few other factors have delayed the development of genetically modified sugarcane varieties. For instance, sugarcane mills operate using a considerable number of varieties to ensure feedstock along the harvesting time with their own transformation and regeneration capacity. Moreover, most of the multinational biotechnology companies focus their research on developing genetically modified varieties for major crops cultivated worldwide, which is not the case for sugarcane grown in tropical climates, mainly in developing countries. Such factors have delayed for almost two decades the development of genetically modified sugarcane cultivars in comparison with other relevant crops, like maize, soybean, and cotton. Despite these complications, the establishment of genetic transformation protocols and reporter/selection genes, have demonstrated *Agrobacterium*– mediated and particle bombardment transformation of sugarcane with sufficient efficiency to produce commercial varieties (Birch and Maretzki, 1993; Bower et al., 1996; Birch, 1997; Irvine and Mirkov, 1997; Arencibia et al., 1998; Elliott et al., 1998, 1999; Enríquez-Obregón et al., 1998; Joyce et al., 1998a, b; Allsopp et al., 2000).

The development of reliable transformation protocols allowed the introduction of several genes of commercial interest into sugarcane genome, transferring traits as herbicide tolerance, resistance to diseases and pests, tolerance to drought, and increased sucrose content (Falco et al., 2000; Braga et al., 2003; Molinari et al., 2007; Zhang et al., 2015; Table 1). Besides the evident interest in changing sucrose metabolism (Botha and Black, 2000; Rossouw et al., 2007, 2010; Zhang et al., 2015; Ma et al., 2020), sugarcane has also been transformed with genes aiming the use of the plant as a biofactory, producing high-value products such as bioplastics (Lakshmanan et al., 2005) and isomers of sucrose (Wu and Birch, 2007). So far, attempts to identify and utilize potential targets for sugarcane improvement relied on genomic and transcriptomic data obtained either experimentally or *in silico*. This was the case for a sugarcane dirigent-like jacalin (*ShDJ*), a transcription factor (*ShSHN1*), and a laccase (*SofLAC*), whose functions were successfully investigated. *ShDJ* conferred higher water stress resistance and improved saccharification in rice plants (Andrade et al., 2019), SofLAC was related to the lignification process (Cesarino et al., 2013) and ShSHN1 was involved in secondary CW formation and enhanced saccharification (Martins et al., 2018).

**TABLE 1:** Examples of markers and traits engineered into sugarcane genome updated from Hotta et al. (2010).

Trait	Gene	Transformation method	References
**Reporter and selection systems**			
Neomycin phosphotransferase	*nptII*	Microprojectile	Bower and Birch, 1992
		Microprojectile	Bower and Birch, 1992
		Microprojectile	Joyce et al., 2014
		*Agrobacterium*	Joyce et al., 2014
β-Glucuronidase	*uidA*	Microprojectile	Noguera et al., 2015
		Electroporation	Arencibia et al., 1995
		*Agrobacterium*	Arencibia et al., 1998
Hygromycin phosphotransferase	*hpt*	*Agrobacterium*	Arencibia et al., 1998
Green fluorescent protein	*gfp*	*Agrobacterium*	Elliott et al., 1998
Phosphinothricin acetyl transferase	*bar*	*Agrobacterium*	Elliott et al., 1998
Phosphinothricin acetyl transferase	*bar*	*Agrobacterium*	Manickavasagam et al., 2004
**Herbicide resistance**			
Bialaphos	*bar*	Microprojectile	Gallo−Meagher and Irvine,1996
Phosphinothricine	*bar*	*Agrobacterium*	Enríquez-Obregón et al., 1998
		Microprojectile	M. C. Falco et al., 2000
		Microprojectile	Gao et al., 2016
Glufosinate ammonium	*pat*	Microprojectile	Leibbrandt and Snyman, 2003
Glyphosate	*EPSPS*	Microprojectile	Noguera et al., 2015
		*Agrobacterium*	Wang et al., 2017
		*Agrobacterium*	Cristofoletti et al., 2018
**Disease resistance**			
SCMV	*SCMV-CP*	Microprojectile	Joyce et al., 1998a, b
			Yao et al., 2017
			Aslam et al., 2018
Sugarcane leaf scald	*albD*	Microprojectile	Zhang et al., 1999
SrMV	*SrMV-CP*	Microprojectile	Ingelbrecht et al., 1999
		*Agrobacterium*	Guo et al., 2015
*Puccinia melanocephala*	Glucanase, chitanase and *ap24*	*Agrobacterium*	Enríquez et al., 2000
Sugarcane yellow leaf virus	*SCYLV-CP*	Microprojectile	Rangel et al., 2003
			Gilbert et al., 2009
Fiji leaf gall	FDVS9 ORF 1	Microprojectile	McQualter et al., 2004b
*Colletotrichum falcatum*	β-1,3-glucanase	*Agrobacterium*	Nayyar et al., 2017
**Pest resistance**			
Sugarcane stem borers	*cry1A*	Electroporation	Arencibia et al., 1999
	*cry1Ab*	Microprojectile	Braga et al., 2003
		*Agrobacterium*	Wang et al., 2017
		*Agrobacterium*	Cheavegatti-Gianotto et al., 2018
	*cry1Ac*	Microprojectile	Gao et al., 2016
		*Agrobacterium*	Cheavegatti-Gianotto et al., 2019
	*cry1Ab* and *cry2Ab*	*Agrobacterium*	Cristofoletti et al., 2018
Sugarcane canegrub resistance	*gna* and *pinII*	Microprojectile	Nutt et al., 1999
Mexican rice borer	*gna*	Microprojectile	Legaspi and Mirkov, 2000
Sugarcane stem borer and Mexican rice borer	*gna*	Microprojectile	Sétamou et al., 2002
**Stress tolerance**			
Drought tolerance	*AtDREB2A CA*	*Agrobacterium*	Souza et al., 2019b
	*AtBI-1*	Microprojectile	Ramiro et al., 2016
**Metabolic engineering/alternative products**			
Sucrose metabolism	Antisense soluble acid invertase	Microprojectile	Ma et al., 2000
	Soluble acid invertase	Microprojectile	Botha et al., 2001
	Antisense neutral invertase	-	Rossouw et al., 2007, 2010
	*manA*	Microprojectile	Zhang et al., 2015
Biomass digestibility	*COMT*	Microprojectile	Jung et al., 2012, 2013
	*SacBAHD01*	*Agrobacterium*	Souza et al., 2019c
Fructo oligosaccharide	*lsdA*	*Agrobacterium*	Enríquez et al., 2000
Polyphenol oxidase	*ppo*	Microprojectile	Vickers et al., 2005
Polyhydroxybytyrate	*phaA*, *phaB* and *phaC*	Microprojectile	Brumbley et al., 2003
		Microprojectile	Petrasovits et al., 2012
ρ-Hydroxybenzoic acid	*hchl* and *cpl*	Microprojectile	McQualter et al., 2004a
Tripsin inhibitors	Kunitz and Bower-Birch	Microprojectile	Falco and Silva-Filho, 2003
Mannose	*manA*	Microprojectile	Jain et al., 2007
Isomaltulose	*SI*	Microprojectile	Wu and Birch, 2007
	*Ims*	*Agrobacterium*	Basnayake et al., 2012
Proline production	*P5CS*	Microprojectile	Molinari et al., 2007

Any transgenic sugarcane variety produced by DNA recombinant technology must undergo an extensive biosafety assessment by worldwide regulatory authorities to ensure environmental and food/feed safety to enter the market. Indonesia and Brazil are both examples of countries where genetically modified sugarcane varieties can be commercially feasible. Research efforts are now focused on achieving predicted and more precise changes to the sugarcane genome, which could be a solid strategy to target changes in C allocation as well as the introduction of synthetic metabolic routes for the production of high-value chemicals. Jung et al. (2016) applied an intragenic precision breeding strategy for RNAi suppression of the 4-coumarate:CoA ligase (4CL), a key enzyme in the phenylpropanoid pathway not only for the production of lignin but also other compounds such as stilbenes and flavonoids. Sequence analysis of sugarcane and closely related species like *Sorghum bicolor* enabled the identification of two isoforms and the functional characterization suggested that *Sh4CL1* was the key component for lignin biosynthesis. Field trials with *Sh4CL1* RNAi sugarcane transgenic line resulted in up to 76% improved saccharification efficiency of lignocellulosic biomass compared to wild type, endorsing the capacity of intragenic precision breeding strategy to targeted redirect metabolic routes.

More recently, genome editing protocols are creating new opportunities to improve crop genomes. These techniques can relieve the current regulatory burden over genetic modified organisms due to its potentially more precise changes in plant genome. However, the application of these technologies to polyploid species has yet to face challenges such as the need for designing guide RNAs targeting all gene copies of homologous genes (paralogs and orthologs) simultaneously while avoiding non-specific (off-target) regions (Zaman et al., 2019). Therefore, it is no surprise that there is just a single published report on the successful use of genome editing in sugarcane. Jung and Altpeter (2016) deployed transcription activator-like effector nuclease (TALEN) to induce mutations in a highly conserved region of the caffeic acid O-methyltransferase (COMT), a key enzyme in the monolignols biosynthesis. The authors were able to demonstrate that 8–99% of the wild type COMT was converted into mutant COMT in different events and that these mutation frequencies were positively correlated with reduced lignin content. Although this is the only data on genome editing in sugarcane so far, it suggests that the use of this technology in sugarcane can be a viable alternative.

## Concluding Remarks

Sugarcane breeding strategies have long focused on increasing sucrose content and improving stress resistance. The emerging interest in sugarcane biomass, either for 2G or green chemicals, widened the desired breeding goals and the demand on molecular phenotyping. Depicting the molecular physiology of the sugarcane control on C partition and storage, as the regulation of fiber (CW) and sugar partition in the culm, can provide feasible ways to optimize C potential.

The sequencing of the ancestors and modern cultivars genomes can enhance the efficiency of biotechnology tools to target specific allelic and regulatory regions. The advance of molecular breeding approaches for polyploids, which combine QTL, GWAS, and GS models with allele dosage information are promising to provide a better incorporation of molecular markers into breeding programs and foster the selection of new cultivars. On the other hand, notable genetics and omics initiatives pointed out several targets as the ones involved in sugar metabolism (i.e., SPS, SWEET, SuSy) or reduced CW recalcitrance (lignins, GHs, Prx, and LAC) as alternatives to improve product yield. Furthermore, progress of analytical tools has been allowed a broader coverage of vast number of compounds, which differ in their chemical and biological properties. Such approach opens a great perspective not only for the investigation of biochemical pathways and their regulatory networks for the better use of C, but also identification of biomarkers. Finally, sugarcane genetic transformation arrives as a feasible alternative to speed up classical breeding, successfully used for improving stress resistance, increasing sucrose, and rising saccharification rates. Despite being modestly employed in this crop, genome editing is presented as a promising tool. Panoramic approaches as expression atlas and co-expression networks can point out new markers and traits associated with C gain improvements and distribution into valuable compounds that are emerging in the context of sugarcane use as a biorefinery.

Altogether, the development of transformation protocols, the increasing knowledge of sugarcane genome, the discovery of new target genes, and the release of genetically modified sugarcane into the market have been paving the way to develop even better new sugarcane varieties that unlock sugarcane C potential in the mid-term.

## Author Contributions

All authors listed have made a substantial, direct and intellectual contribution to the work, and approved it for publication.

## Conflict of Interest

The authors declare that the research was conducted in the absence of any commercial or financial relationships that could be construed as a potential conflict of interest.

## Footnotes

1 RAIZEN (2020) https://www.raizen.com.br/en/our-business/ethanol (Accessed June 13, 2020).

2 BioRender (2020). biorender.com (Accessed November 26, 2020).

3 eRcane in Réunion; http://www.ercane.re/ (2020) (accessed July 17, 2020).

4 Instituto Agronômico de Campinas -IAC (2020) http://www.iac.sp.gov.br/ (Accessed July 20, 2020).

5 Rede Interuniversitária para o Desenvolvimento do Setor Sucroenergético - RIDESA (2020). https://en.ridesaufscar.com.br (Accessed October 3, 2020).

## Glossary

**Abbreviations:** 1G, first generation ethanol; 2G, second generation ethanol; C4H, Cinnamate-4-hydroxylase; 4CL, 4-Coumarate:CoA Ligase; BACs, Bacterial Artificial Chromosome; CDS, Coding Sequences; COMT, Caffeic acid O-methyltransferase; CW, Cell Wall; EXPAs, Expansins; GHs, Glycosyl Hydrolases; GS, Genomic Selection; GTs, Glycosyl Transferases; GWA, Genome Wide Association Studies; DArT, Diversity Arrays Technology; EST, Expressed Sequence Tags; Hex, Hexose; Inv, Invertase; LAC, Laccase; LD, Linkage Disequilibrium; LFY, Leafy; LGN, Lignin; MTAs, Marker-Trait Associations; OR, Oxidoreductases; PACs, Proteins related to carbohydrates metabolism; PAL, Phenylalanine Ammonia-Lyase; PHB, Polyhydroxybutyrate, PHYC, Phytochrome C; PLT, Polyol Transporter; Prx, Peroxidases; NUE, Nitrogen Use Efficiency; QTL, Quantitative Trait Locus; SDM, Single-Dose Markers; SNPs, Single Nucleotide Polymorphisms; Suc, Sucrose; SuSy, Sucrose Synthase; SPP, Sucrose Phosphate Phosphatase; SPS, Sucrose Phosphate Synthase; ST, Sugar Transporters; SWEET, Sugars Will Eventually be Exported Transporters; TAG, triacylglycerol; TALEN, transcription activator-like effector nuclease; TF, Transcription Factors; TOR, Target of Rapamycin; TSTs, Tonoplast Sugar Transporters; UDP, Uridine Diphosphate Glucose; Vac, Vacuolar.

### Bagasse

The residual solid biomass generated after the broth, rich in sucrose, extracted from sugarcane. Its composition comprises lignin, cellulose, and hemicellulose.

### Genotyping-by-Sequencing (GBS)

Rapid and low-cost DNA sequencing technique based on the massive use of restriction enzymes to efficiently discover and map single nucleotide polymorphisms (SNPs) in large genomes.

### Genotype × Environment (GxE) Interaction

The interaction between a specific genotype in a specific environment or set of environmental conditions, resulting in a distinct phenotype.

### Genome-Wide Association Studies (GWAS)

Large-scale observational studies linking genomic variations with particular traits in genetically diverse populations, attempting to identify targeted genetic markers to a specific trait.

### Linkage Disequilibrium (LD)

The dependent association of gene variants at two or more loci observed in a population.

### Molasses

The dark viscous waste product resulting from the refinement of sugarcane broth. It has a high concentration of reduced sugars and sucrose.

### Quantitative Trait Locus (QTL)

A genomic region associated with a trait, based on which markers linked to a specific phenotype are developed.

### Recalcitrance

The resistance of sugarcane biomass to fermentation, mostly caused by cell wall component lignin, adding more complexity to sugar production and refining.

### Saccharification

The set of catabolic reactions that breaks down polysaccharides such as starch or cellulose, generating sucrose for sugar production or monosaccharide molecules for ethanol production.
